# Inequality in modern contraceptive use and unmet need for contraception among women of reproductive age in Zambia. A trend and decomposition analysis 2007–2018

**DOI:** 10.1186/s12978-024-01909-8

**Published:** 2024-12-09

**Authors:** Joseph Kazibwe, Felix Masiye, Marie Klingberg-Allvin, Björn Ekman, Jesper Sundewall

**Affiliations:** 1 Department of Clinical Science, Lund University, Malmö, Sweden; 2 Department of Economics, University of Zambia, Lusaka, Zambia; 3 Department of Women and Childrens Health, Karolinska Institutet, Stockholm, Sweden; 4 HEARD, University of KwaZulu-Natal, Durban, South Africa

**Keywords:** Modern contraceptive use, Unmet need for contraception, Inequality, Decomposition of inequality, Low- and middle-income Countries, Zambia

## Abstract

**Background:**

Access to contraception can be a transformational intervention towards advancement of education, health, and freedom of choice. Countries have committed to improving access to contraception enshrined in the sustainable development goals (SDGs), indicator 3.7.1. Our study seeks to investigate the level of inequality in current use of modern contraception and unmet need for contraception among sexually active women of reproductive age in Zambia during 2007, 2013/14 and 2018 to inform family planning policy.

**Methods:**

We use three rounds of Zambia demographic and health survey datasets for the years 2007, 2013/14 and 2018, which are nationally representative surveys. We included a total of 19,973 sexually active women of reproductive age from 15 to 49 years living in Zambia. The level of inequality was assessed using concentration curves, and indices. The concentration indices were decomposed to identify the causes of the inequality.

**Results:**

Our analysis shows that there was inequality in the current use of modern contraception across the years 2007, 2013/14 and 2018. The concentration curves showed that current use of modern contraception was higher among the wealthy than the poor. This pro-rich trend was consistent throughout the study period. Erreygers concentration Index (EI) values were 0.2046 in 2007, 0.1816 in 2013/14, and 0.1124 in 2018. The inequality in current use of modern contraception was significantly influenced by having access to contraceptive counselling, education level and being in a union (living with a partner). In addition, there was inequality in unmet need for contraception with concentration curves showing that unmet need for modern contraception was experienced more among the poor compared to the wealthy. Unmet need was thus pro poor. The EI values were – 0.0484 in 2007, – 0.0940 in 2013/14 and – 0.0427 in 2018. This inequality was significantly influenced by education, employment status, being in a union, and having health insurance.

**Conclusion:**

Inequality in modern contraceptive use and unmet need for contraception exists and has persisted over the years in Zambia. Such inequality can be addressed through a multipronged approach that includes encouraging women to visit health facilities, access to contraceptive counselling, and promoting formal education.

**Supplementary Information:**

The online version contains supplementary material available at 10.1186/s12978-024-01909-8.

## Plain Language Summary

**Background to the study:**

The use of effective methods of contraception (also known as modern contraception) has several advantages including prevention of dropping out of school due to unintended pregnancies, increased freedom to choose when to have a child, among others. Such advantages translate into development of the community and society at large. Increasing access to contraception is one of the targets under the sustainable development goals. The level of inequality in contraceptive use affects the chances of achieving the target. Our study therefore aims to find out whether there are any existing inequalities in modern contraceptive use among women in Zambia that still have the capacity to give birth (from 15- to 49-yearolds).

**Methods used in this study:**

The study looks at two measures, using modern contraception and unmet need for contraception. Unmet need for contraception in this study is where a woman that is sexually active and does not want to get pregnant, but she is not using any form of contraception. We use information that has been collected in a way that it represents the population of Zambia. The information was collected at three time points, that is 2007, 2013/14, and 2018. A total 19,973 women provided the information. We investigate the presence of inequalities through graphs called concentration curves and estimate the quantity of inequality. We further use the latest and reliable method by find what causes the inequality.

**Results of the study:**

We find that there are inequalities in modern contraceptive use and unmet need for contraception based on population-based data collected during 2007, 2013/14, and 2018 in Zambia. The inequalities favour the rich people in society where you find rich people using modern contraception more than the poor people. For the inequality in unmet need for contraception, which is a negative indicator, we found that it is the poor people that experience unmet need for contraception compared to the rich people. The causes of these inequalities included having no formal education, employment status, being in a union, and having health insurance.

**Conclusion of the study:**

There is inequality in the use of modern contraceptives and unmet need for contraception in Zambia. This inequality can be addressed through multiple ways including encouraging women to visit health facilities, access to contraceptive counselling, promoting formal education among others.

**Supplementary Information:**

The online version contains supplementary material available at 10.1186/s12978-024-01909-8.

## Resumen

**Antecedentes:**

El acceso a la anticoncepción puede ser una intervención transformadora hacia el avance de varios factores del capital humano, incluida la educación, la salud y la libertad de elección, la comunidad y el desarrollo social. Los países se han comprometido a mejorar el acceso a la anticoncepción consagrado en los Objetivos de Desarrollo Sostenible (ODS), indicador 3.7.1. Este estudio investiga el nivel de desigualdad en la utilización y las necesidades insatisfechas de anticonceptivos modernos entre mujeres sexualmente activas en edad reproductiva en Zambia de 2007 a 2018 para informar la política de planificación familiar.

**Métodos:**

Se utilizó tres rondas de datos de la Encuesta Demográfica y de Salud de Zambia para los años 2007, 2013/14 y 2018. Las encuestas son representativas a nivel nacional. Se incluyó un total de 19,973 mujeres sexualmente activas en edad reproductiva de 15 a 49 años que vivían en Zambia. El nivel de desigualdad se ilustró mediante curvas de concentración, que se estimaron y descompusieron utilizando el último método de Heckley et al.

**Resultados:**

La desigualdad en el uso de anticonceptivos modernos fue pro-rica a lo largo de los años 2007, 2013/14 y 2018: índice de concentración de Erreygers (IE) 0,2046, 0,1816 y 0,1124 respectivamente. La desigualdad en el uso de anticonceptivos modernos estuvo influenciada por el acceso al asesoramiento sobre anticonceptivos, el nivel educativo y el hecho de estar afiliados a un sindicato. La desigualdad en la necesidad insatisfecha de anticonceptivos fue pro-pobre: IE de -0,0484 en 2007, − 0,0940 en 2013/14 y − 0,0427 en 2018. Esta desigualdad estuvo influenciada por la educación, la situación laboral, estar afiliado a un sindicato y tener seguro médico.

**Conclusiones:**

La desigualdad en el uso y la necesidad insatisfecha de anticonceptivos modernos existen y han persistido a lo largo de los años en Zambia. Esta desigualdad puede abordarse mediante un enfoque múltiple que incluya alentar a las mujeres a visitar los centros de salud, acceder al asesoramiento sobre anticonceptivos y promover la educación formal.

## Introduction

Access to contraception is linked to the advancement of several personal attributes considered essential for economic development, including education, health, and freedom of choice [1]. It also has positive economic implications at individual, the community and national levels [2]. Contraception can be defined as the use of a device, medication, procedure or behavior to avoid getting pregnant or space pregnancies [3]. Contraceptive methods can be categorized into modern and traditional methods. The use of contraception enables individuals the ability to plan and space births reducing pregnancy related risks for women, consequently reducing maternal and infant mortality [1, 4]. Contraception enables young girls to delay getting pregnant thus avoiding unsafe abortions, increasing chances to further their education and get gainful employment [2, 5]. Modern contraception is a highly cost effective intervention [6] and is part of the sustainable development goal (SDG) 3: Indicator 3.7.1 which focuses on ensuring universal access to sexual and reproductive health-care services, including contraceptive use, by 2030 [7].

Many countries have made progress regarding access to and utilization of contraception. The use of modern contraception globally has increased from 35% in 1990 to 45% in 2021 among women [8]. Despite this improvement, the desired goal of universal access to contraception is still far from reach with over 164 million women of reproductive age reporting unmet need for contraception [8]. The majority of the unmet need for contraception is found in sub–Saharan Africa (SSA) [8]. It has been predicted that the increase in modern contraceptive use by two percent a year can reduce maternal mortality to a tune of approximately 67% based on estimates in a total of 88 most burden countries when used with other maternal related interventions [9].

One of the main threats to universal access to contraception is inequality in contraceptive access and use. Contraceptive use is considered a health behaviour and therefore inequality in contraceptive use can be considered a health inequality. The causes of health inequalities are complex, diverse, changing and interlinked [10]. These factors should be analysed when assessing inequality in contraceptive use and unmet need for contraception. Health inequalities can be explained through different models and theories including the social determinants of health [11], social ecological model and, the social cognitive theory (SCT) [12]. The social determinants of health are individual factors and conditions within the environment we live that have an influence on our health. These include individual and lifestyle factors, socioeconomic, cultural, and environmental conditions. For example, age, sex, marital status, education level, employment status and income [13]. Having differences in these factors may consequently lead to inequalities in health behaviour and outcomes including contraceptive use. The social determinants of health model can be further expounded by the social ecological model which emphasizes the interaction between individual, interpersonal, institutional, community and policy factors [14, 15]. In addition to social determinants of health and the social ecological model, health behaviour can be explained by the SCT. SCT stipulates that current behaviours, thoughts and emotions, and environment all interact to affect new behaviour [12]. The interaction of the factors and their evolution over time could explain the existing inequalities in contraceptive use.

The existence of inequality in contraceptive use among women has been linked to a person’s education level, type of employment and wealth [16–19]. Education increases a woman’s knowledge and awareness about contraception, available methods, its importance, debunking misconceptions, and myths surrounding contraceptive use. This increases willingness to use contraceptives [20, 21]. In addition, educated women are more likely to be employed where one’s fertility control for example child spacing and planning is necessary to avoid employment complications and career stagnation. Further, the type of employment is reported to have a bearing on the choice of contraception [22]. The influence of education on the inequality in contraceptive use among women may vary by setting (urban and rural) [16]. Wealth status is positively associated with contraceptive use [23, 24]. Women with a higher wealth status have financial capacity to overcome financial barriers to access contraception while the poorer people may not [25]. This contributes to inequality in contraceptive use [16–19].

Our study aims to investigate the level of inequality in current use of modern contraception and unmet need among sexually active women of reproductive age in Zambia in 2007, 2013/14, 2018, and consequently identify causes of the inequality. Zambia is one of the countries in SSA with the highest increase in the use of modern contraception among women of reproductive age [8]. This is partly due to efforts to boost modern contraceptive use for example deployment of community health workers, dissemination of family planning messages, offering family planning counselling and inclusion of contraception in the health benefit package for the National Health Insurance Scheme [26, 27]. However, the achieved increment in contraceptive use is far from the national targets of at least 70% of married women using some form of contraception and 60% using a modern contraceptive method by 2026 [28]. The proportion of women using modern contraception in Zambia as of 2018 is only 48% [29]. There is still a considerable level of unmet need for modern contraception (20%) [29] and the population continues to increase exponentially [30]. Stipulated in its 2022–2026 National Health Strategic Plan, Zambia seeks to increase contraceptive use among women as an essential intervention to reduce the maternal mortality ratio, manage rapid population growth, and stimulate economic development [31]. To enable Zambia reach the set contraceptive use target, understanding the magnitude and influencers of inequalities that exist in contraceptive use in the country is important. Such information can help identify the disadvantaged population sub-groups and guide development of interventions to tackle unmet need for contraception. The findings of this study are also relevant to similar countries and to the broader research community as they show how inequalities can be assessed using readily available data.

The majority of inequality in contraceptive use is socioeconomic and preventable, but this requires evidence which is currently lacking. Studies on contraceptive use have so far mostly focused on determining whether there are differences in utilization among different population sub groups or and identify contributors to the differences [21, 32–34]. This makes it difficult to compare the level of socioeconomic inequality between different disease or health service areas and over time. In contrast, quantifying the inequalities in one measure (index) makes the comparison possible. No study has quantified the level of socioeconomic inequality in modern contraceptive use or unmet need for contraception in Zambia, which is important to understand how inequality is distributed, how this has changed over time and what the main drivers are. A number of studies have been conducted in other SSA countries. A study from Benin, along with a multi-country study (using data from 47 sub-Saharan countries including Zambia) by Budu et al [18] and Fentie et al [17] respectively revealed that there is inequality in modern contraceptive use. Women in wealthier quintiles disproportionately use modern contraception more than women in poorer or less wealthy quintiles. Budu et al. reported that the inequality was influenced by the woman’s age group, parity, residential setting (rural or urban), ethnicity, exposure to media and education level [18]. While Fentie et al. found that the inequality was mostly driven by residential setting (urban/rural), marital status, distance to the health facility and exposure to media. However, both studies utilized the Wagstaff, Doorslaer, and Watanabe (WDW) method of inequality decomposition [35] which has been criticized for having several weaknesses [36]. For our study we use a recent and more robust method by Heckley et al. [36] In addition, the earlier studies included women of reproductive age while our study included currently sexually active women among the more general group of women of reproductive age. The rationale for adopting this approach is that they are particularly at risk of experiencing an unwanted pregnancy [37, 38]. For example, in Zambia, approximately 45% of all pregnancies are reported to be unintended as of 2018 [39] and thus our intention is to focus on women that are most likely to be using contraception for birth control or child spacing.

Therefore, investigating the level of inequality in current use of modern contraception and unmet need for contraception is essential in understanding the key drivers and causes of the inequality which is crucial to informing appropriate contraceptive use policy. We use a nationally representative population-based sample to quantify and decompose inequalities in current use of modern contraceptive and unmet need for contraception.

## Methods

### Study design

This is a repeated cross-sectional population based study and follows the Strengthening the Reporting of Observational studies in Epidemiology (STROBE) guidelines [40].

### Setting

The study was carried out in Zambia, a lower middle-income country located in the southern part of Africa.

### Participants

The study included 19,973 sexually active women of reproductive age from 15 to 49 years living in Zambia. A woman was considered sexually active if they had had sex at least once in the last 30 days from the time of the interview.

### Variables

#### Dependent variables

There are two dependent variables.

- Current use of modern contraception
- Unmet need for contraception

Current use of modern contraception is the current use of any of the following methods: female sterilization, contraceptive pill/oral contraceptives, intrauterine contraceptive device (IUD), injectables, implants, female condom, male condom, diaphragm, contraceptive foam, and contraceptive jelly, lactational amenorrhea method (LAM), standard days method [41]. Current use of modern contraception is a binary variable based on a question that asked the study participants to share the types of contraception that they were currently using. Using the responses, the respondents were identified as currently using modern contraception or not.

**Fig. 1:**
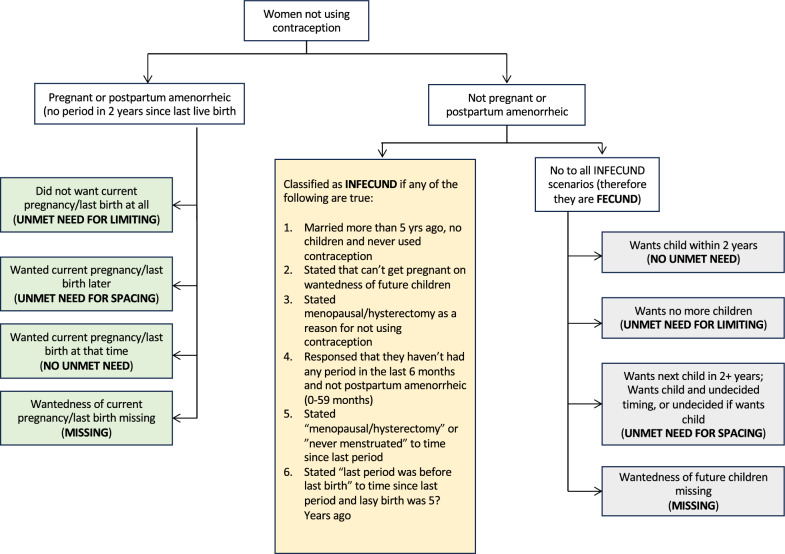
Diagram showing determination of unmet need for contraception

Unmet need for contraception is a situation where a woman does not want to get pregnant but is not using contraception during sexual intercourse [42]. Unmet need for contraception is a binary variable ascertained using 15 questions [43, 44] based on which the unmet need for spacing and unmet need for limiting are determined by the DHS. Unmet need for spacing refers to a situation where a woman wants to have a child in the future but not at the present time, and is not using contraception during sexual intercourse. Unmet need for limiting describes a situation where a woman does not want to have any more children but is not using contraception during sexual intercourse. If a participant experiences either of the two (unmet need for limiting or unmet need for spacing), then they are categorised as having unmet need for contraception. Below is the flow chart (Fig. 1) showing the categorisation of the unmet need for contraception.

Figure 1 is an author generated illustration based on the “Revising unmet need for contraception” report [44].

#### Independent variables

The selection of the independent variables were identified by Kazibwe et al [45] and informed by social determinants of health, social ecological model and SCT. The variables included demographic characteristics of the woman, household characteristics, partner characteristics, community characteristics and perceptions. The specific variables are wealth status, age group, highest level of education, religion, sex of household head, employment status (currently working), currently in a union (currently staying with a partner), exposure to family planning messages through media, contraceptive counselling by health worker (counselling done by health worker either at health facility or during community outreach), health insurance coverage and place of residence (rural/urban). The participants’ wealth status is determined by the wealth index as calculated by the DHS [46].

### Data sources

This study utilises three rounds of cross-sectional Zambia demographic and health surveys (ZDHS) analysed independently. The surveys are ZDHS 2007, ZDHS 2013-14, and ZDHS 2018. The ZDHS is nationally representative survey carried out by the Zambia Statistics Agency and the Ministry of Health supported by cooperating partners. The ZDHS was the preferred data source for this analysis because it captures population based data on maternal and reproductive health services utilisation including the variables that are relevant to this study. In addition, the ZDHS survey has a standardized questionnaire that has been validated to produced high quality data on a population level for the last 30 years.

### Study size and bias

The ZDHS follows a stratified two-stage sample design. The first stage involves selecting sample points/clusters consisting of enumeration areas. The enumeration areas are selected with a probability proportional to their size within each sampling stratum. The second stage involves systematic sampling of households with household listing done in all the selected clusters. This sampling design allows the sample to be representative at the national and provincial levels and among the urban and rural areas. The sampling design reduces chances of bias. The datasets cover vast areas on reproductive, maternal and child health and nutrition. The datasets contain relevant variables to our study covering contraceptive use [29].

Of the 19,973 participants in total included in this study, 18.9% (3,779) of the participants were from ZDHS 2007, 45.1% (9,010) of the participants were from the ZDHS 2013/14 and 36% (7,184) participants were from ZDHS 2018. We tested the homogeneity of the three datasets (ZDHS 2007, ZDHS 2013-14, and ZDHS 2018) using the Bartlett’s equal-variances test to test the hypothesis.

### Analysis

The analysis included three steps. (a) concentration curves and corrected concentration index (CCI), (b) CCI decomposition and (c) analysis of trends over time.

- Concentration curves and CCI
  Inequality in modern contraceptive use and unmet need for contraception was measured using the concentration curves and concentration index. The concentration curve is a graphical representation of how benefits such as utilisation of a health service are distributed across different socio-economic groups or categories. The concentration curve plots the cumulative proportion of one outcome variable against the cumulative proportion of the population ranked by another variable, in this case the wealth index [47]. The wealth index is a continuous variable in our study as estimated by DHS. It is a composite measure constructed to reflect the household’s cumulative living standard. Responses to questions in the questionnaire eliciting the ownership of specific household assets or effects are used to construct the index through a statistical procedure called principal components analysis. Some items asked about are household effects, means of transportation, agricultural land, and livestock/farm animals [48]. The distribution of wealth status is available in supplement 1.
  We used the concentration curves to identify whether socioeconomic inequality in each outcome variable exists and how pronounced it is at each point in time. The concentration curve has the cumulative percentage of the health variable on the y axis and the cumulative percentage of the population ranked by the wealth index on the x axis beginning from the poorest to the richest. The curve has a 45-degree line also known as the line of equality that starts at the intersection of the x and y axes (bottom left corner). If the concentration curve is above the line of equality then the health variable is pro poor and if the curve is below the line of equality then the health variable is pro rich [49].
  The concentration index (CI), a bivariate rank dependent index, is defined as twice the area between the concentration curve and the line of equality (the 45-degree line), was used quantify the degree of inequality that exists for a given outcome variable. The index ranges from -1 to 1 that is, it can be zero, positive or negative implying equal benefit by both the rich and poor, poor benefit more and lastly rich benefit more respectively [47]. Equations on the derivation of a CI can be found in for example Kakwani [50], Lerman &Yitzhaki [51] and Jenkins [52].
  Considering that our outcomes of interest are bound binary variables (0,1), the estimates of CI are likely to be inaccurate because the bounds of the CI may depend upon the mean of the health variable making it difficult to compare populations with different mean health levels [53, 54]. The mean of the distribution of a binary variable places bounds on the possible values of the CI where if the mean increases, the range of the possible values the CI can take shrinks [54]. Further, different rankings will be obtained depending on the health variable under consideration. The ranking obtained when considering inequalities in ill health will be different from the rankings obtained when considering inequalities in health [53].
  Therefore, a corrected concentration index (CCI) also known as the Erreygers concentration index (EI) was used for this study. The concentration curve and index were estimated using the conindex [47] commands in Stata 17. The clusters and strata of the samples were adjusted for in the estimation of the EI by including pweights in the syntax.
- CCI decomposition
  In the decomposition, we sought to identify the causes of the existing inequality in the outcome variables and estimate the contribution of the independent variables towards the inequalities in the outcome variable of interest [55]. We opted for the general method for decomposing the causes of socioeconomic inequality in health described by Heckley et al. to identify the causes of the inequality [36]. Heckley et al. argue that their method for decomposition of inequalities is more suitable in determining the causal effect of a covariate on the inequality index [36]. The decomposition method by Wagstaff et al. has some important limitations. It is one dimensional focusing on health and ignoring the rank based on the socioeconomic variable. Therefore, the decomposition method by Wagstaff explains only the variation of the health variable but not the covariance between the health variable and rank based on socioeconomic status [36]. Therefore, WDW does not determine the causes of the inequality. Other challenges of the decomposition method by Wagstaff et al. include the difficulty to interpret the results. The general method for decomposing the causes of socioeconomic inequality in health shows marginal contributions, making it more useful to policymakers who seek to understand the key drivers of inequality in contraceptive coverage.
  The general method for decomposing the causes of socioeconomic inequality in health can be considered more reliable as it applies regression of recentered influence function (RIF). Therefore, to estimate the marginal effects of the covariates on the index, the method follows two steps. (1) computing the RIF of the rank dependent index, and (2) regressing the RIF on a set of covariates. The RIF regression estimates the marginal contributions that the different covariates have on the inequality index.
  In decomposition, we started with decomposing the Erreygers concentration Index (EI) for both modern contraception and unmet need using the RIF-EI-OLS decomposition method. This was followed by decomposing the different types of concentration indices including concentration index (CI), attainment relative concentration index (ARCI), Shortfall relative concentration index (SRCI), Wagstaff Index (WI) using the RIF-I-OLS method where I stands for the name of the respective index.
  The RIF -I-OLS decomposition findings can be interpreted based on the estimated coefficient of a given covariate. The coefficient is the association been the covariate and the influence on the index [36].
- Description of trends over time
  We described the trends of the CCI for each outcome variable over time (from 2007 to 2018). This involved identifying changes in inequality that occurred over time thus revealing the trajectory of inequality in modern contraceptive use and unmet need for contraception.

## Results

### Characteristics of the study participants

Table 1 shows the characteristics of the study participants categorized into contraceptive use variables and demographic characteristics across the years 2007, 2013/14 and 2018. There was a gradual increase in the prevalence of using modern contraception from 37.26 percent in 2007 to 49.75 percent in 2018. The prevalence of unmet need for contraception increased from 15.27 percent in 2007 to 21.31 percent in 2013/14 but later reduced to 19.78 percent in 2018. Majority of the participants in each of the years (2007, 2013/14 and 2018) were between the ages 20–34 years, that is 63.16 percent in 2007, 58.16 percent in 2013/14, and 56.62 percent in 2018. Majority of the participants were from the rural area (64.73–56.28 percent). Primary school was the highest level of education for most of the participants (58.24–51.39 percent). More than 50 percent of the participants were working at the time of the interview across the years. The variance of the demographic variables is generally different across the three samples except for highest level of education.

**Table 1:** Characteristics of the study participants for the years 2007, 2013/14 and 2018

Year	2007		2013/14		2018		Bartlett’s equal-variances test
Variable	Frequency	%	Frequency	%	Frequency	%	*P* value
*Contraception variables*							
*Modern contraceptive use*	(*N* = 3,779)		(*N* = 9,010)		(*N* = 7,184)		**0.043**
No	2,371	62.74	4,813	53.42	3,610	50.25	
Yes	1,408	37.26	4,197	46.58	3,574	49.75	
*Unmet need for contraception*	(*N* = 3,779)		(*N* = 9,010)		(*N* = 7,184)		**0.000**
No	3,202	84.73	7,090	78.69	5,763	80.22	
Yes	577	15.27	1,920	21.31	1,421	19.78	
*Demographic characteristics*							
*Age group*	(*N* = 3,779)		(*N* = 9,010)		(*N* = 7,184)		**0.022**
15–19	339	8.97	767	8.51	610	8.49	
20–24	802	21.22	1,589	17.64	1,399	19.47	
25–29	905	23.95	1,920	21.31	1,394	19.40	
30–34	680	17.99	1,731	19.21	1,275	17.75	
35–39	463	12.25	1,372	15.23	1,120	15.59	
40–44	319	8.44	968	10.74	807	11.23	
45–49	271	7.17	663	7.36	579	8.06	
*Province*	(N = 3,779)		(N = 9,010)		(N = 7,184)		**0.000**
Central	375	9.92	785	8.71	729	10.15	
Copperbelt	395	10.45	905	10.04	746	10.38	
Eastern	539	14.26	1,172	13.01	935	13.02	
Luapula	364	9.63	860	9.54	741	10.31	
Lusaka	448	11.85	998	11.08	875	12.18	
Muchinga	–		802	8.90	662	9.21	
Northern	404	10.69	902	10.01	648	9.02	
North-western	394	10.43	854	9.48	560	7.80	
Southern	483	12.78	1,080	11.99	760	10.58	
Western	377	9.98	652	7.24	528	7.35	
*Place of residence*	(*N* = 3,779)		(*N* = 9,010)		(*N* = 7,184)		**0.004**
Urban	1,492	39.48	3,939	43.72	2,534	35.27	
Rural	2,287	60.52	5,071	56.28	4,650	64.73	
*Highest level of education*	(*N* = 3,779)		(*N* = 9,003)		(*N* = 7,184)		0.307
No education	447	11.83	898	9.97	696	9.69	
Primary	2,201	58.24	4,816	53.49	3,692	51.39	
Secondary	950	25.14	2,827	31.40	2,400	33.41	
Higher	181	4.79	462	5.13	396	5.51	
*Religion*	(*N* = 3,771)		(*N* = 8,986)		(*N* = 7,184)		**0.000**
Catholic	687	18.22	1,523	16.95	1,234	17.18	
Protestant	3,018	80.03	7,364	81.95	5,843	81.33	
Muslim	15	0.40	41	0.46	40	0.56	
Other	51	1.35	58	0.65	67	0.93	
*Sex of head of the household*	(*N* = 3,779)		(*N* = 9,010)		(*N* = 7,184)		**0.006**
Male	3,314	87.70	7,786	86.42	6,211	86.46	
Female	465	12.30	1,224	13.58	973	13.54	
*Currently working*	(*N* = 3,773)		(*N* = 8,973)		(*N* = 7,184)		**0.000**
No	1,729	45.83	3,753	41.83	3,514	48.91	
Yes	2.044	54.17	5,220	58.17	3,670	51.09	
*Wealth statu﻿s*	(*N* = 3,779)		(*N* = 9,010)		(*N* = 7,184)		**0.014**
First Quintile (poorest)	687	18.18	1,595	17.70	1,617	22.51	
Second Quintile	699	18.50	1,846	20.49	1,568	21.83	
Third Quintile	803	21.25	2,048	22.73	1,484	20.66	
Fourth Quintile	909	24.05	1,923	21.34	1,290	17.96	
Fifth Quintile (Wealthiest)	681	18.02	1,598	17.74	1,225	17.05	
*Currently in a union*	(*N* = 3,779)		(*N* = 9,010)		(*N* = 7,184)		**0.000**
No	412	10.90	956	10.61	844	11.75	
Yes	3,367	89.10	8,054	89.39	6,340	88.25	
Exposed to FP messages through media	(*N* = 3,779)		(*N* = 9,010)		(*N* = 7,184)		**0.000**
No	2,118	56.05	5,543	61.52	5,540	77.12	
Yes	1,661	43.95	3,467	38.48	1,644	22.88	
*Accessed contraceptive counselling*	(*N* = 3,779)		(*N* = 9,010)		(*N* = 7,184)		**0.000**
No	2,614	69.17	5,627	62.45	4,523	62.96	
Yes	1,165	30.83	3,383	37.55	2,661	37.04	
*Health insurance coverage*	(*N* = 3,776)		(*N* = 9,003)		(*N* = 7,184)		**0.000**
No	3,497	92.61	8,731	96.98	7,024	97.77	
Yes	279	7.39	272	3.02	160	2.23	

The bold values indicate that the *P* value is less than 0.05

*FP* family planning

**Fig. 2:**
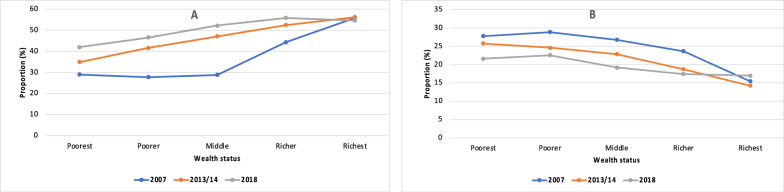
Graph A shows the proportion of women currently using modern contraception per wealth quintile and graph B shows the proportion of women experiencing unmet need for contraception in each wealth quintile in Zambia for the years 2007, 2013/14 and 2018 based on DHS data

Figure 2 contains two graphs; A & B. Graph A shows the proportion of women currently using modern contraception in each wealth quintile over time while graph B shows the proportion of women experiencing unmet need for contraception in each wealth quintile over time. There is generally an increasing proportion of women using modern contraception across all wealth strata over time while the proportion of unmet need for contraception is reducing over time.

### Inequality in current use of modern contraception, 2007-2018

**Fig. 3:**
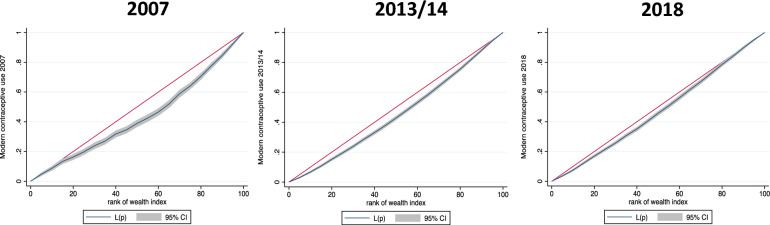
Concentration curves for current use of modern contraception in Zambia for the years 2007, 2013/14 and 2018 using DHS data 45-degree line (red in colour): line of equality

Figure 3 shows three concentration curves for the years 2007, 2013/14 and 2018 showing the extent of inequality in current use of modern contraception among the different wealth ranks. Each concentration curve plots the cumulative percentage of current use of modern contraception on the y axis and the cumulative percentage of the study participants ranked by wealth (based on the wealth index) on the x axis. The study participants are ranked from the poorest to the richest using wealth index. The 45-degree line (line of equality) signifies equal distribution of modern contraceptive use among the rich and the poor. All the three concentration curves have the curve below the line of equality indicating that current use of modern contraceptives is significantly pro-rich where people in higher wealth ranks of society use modern contraception more than those in lower wealth ranks. The difference in the current use of modern methods of contraception across the wealth ranks (the area between the line of equality and the curve) is more prominent in 2007. The concentration curves show signs of gradual decline in subsequent surveys.

### Inequality in unmet need for contraception

**Fig. 4:**
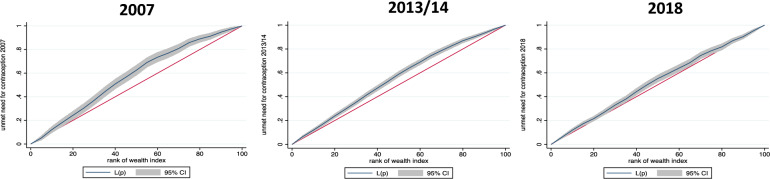
Concentration curves of unmet need for contraception in Zambia for the years 2007, 2013/14 and 2018 using DHS data. 45-degree line (red in colour): line of equality

Figure 4 shows the distribution of unmet need for contraception among the different wealth ranks. It contains three concentration curves for the years 2007, 2013/14 and 2018. Each concentration curve plots the cumulative percentage of unmet need for contraception on the y axis and the cumulative percentage of the study participants ranked by wealth (based on the wealth index; ranked from the poorest to the richest) on the x axis. All curves in the three graphs are above the line of equality. Therefore, the concentration curves show that unmet need for contraception is pro poor reflecting inequality in contraception in all the years. The inequality in unmet need for contraception (the area between the curve and the line of equality) is significantly more profound in 2007 and 2013/14 compared to 2018.

### Erreygers’ Concentration Index (EI)

Inequality in current use of modern contraception as shown in Table 2 displays positive values of the EI. This indicates that women in the wealthier half of the population use modern contraception more than the less wealthy half, in all the years; 2007, 2013/14 and 2018: concentration index 0.2046, 0.1816 and 0.1124 respectively. The inequalities reduce by year from concentration index 0.2046 in 2007 to 0.1124 in 2018. The inequality in modern contraceptive use was significantly influenced by access to contraceptive counselling in 2007; education and being in a union in 2013/14; education level and access to contraceptive counselling in 2018. Access to contraceptive counselling was significantly associated with a reduction of inequalities in modern contraceptive use in 2007 and 2018. Education was also significantly associated with a reduction in the level of inequality of modern contraceptive use in 2013/14 and 2018 where having primary or secondary education reduces inequality in modern contraceptive use. On the other hand, being in a union significantly increased the level of inequality in modern contraceptive use in 2013/14. Women in unions (staying with a partner) were more likely not to use modern contraceptives compared to their peers that were not staying with partners.

**Table 2:** Concentration index (Erreygers Index) for current use of modern contraception and unmet need for contraception in Zambia for the years 2007, 2013/14 and 2018 using DHS data

	Modern contraceptive use									Unmet need for contraception								
	2007 (*N* = 3779)			2013/14 (*N* = 9010)			2018 (*N* = 7184)			2007 (*N* = 3779)			2013/14 (*N* = 9010)			2018 (*N* = 7184)		
	Coeff	95% confidence interval		Coeff	95% confidence interval		Coeff	95% confidence interval		Coeff	95% confidence interval		Coeff	95% confidence interval		Coeff	95% confidence interval	
		Lower bound	Upper bound		Lower bound	Upper bound		Lower bound	Upper bound		Lower bound	Upper bound		Lower bound	Upper bound		Lower bound	Upper bound
CCI (EI)	0.2046**			0.1816*			0.1124***			-0.0484 ***			-0.0940 ***			-0.0427 *		
*Variable*																		
*Age group*																		
15–19	Ref																	
20–24	0.0209	− 0.1227	0.1645	− 0.0932	− 0.2163	0.0299	0.0622	− 0.0518	0.1761	0.0364	− 0.1006	0.1734	0.0262	− 0.0936	0.1460	− 0.0217	− 0.1393	0.0959
25–29	0.0290	− 0.1314	0.1893	− 0.0927	− 0.2126	0.0270	0.0935	− 0.0264	0.2134	0.0276	− 0.1014	0.1564	− 0.0156	− 0.1304	0.0992	− 0.0976	− 0.2058	0.0106
30–34	0.0317	− 0.1058	0.1693	− 0.1025	− 0.2210	0.0161	0.0165	− 0.1048	0.1378	0.0026	− 0.1278	0.1331	− 0.0060	− 0.1196	0.1075	− 0.0800	− 0.1915	0.0320
35–39	0.0603	− 0.1040	0.2245	− 0.0669	− 0.1970	0.0633	0.0495	− 0.0774	0.1763	− 0.0064	− 0.1411	0.1284	− 0.0670	− 0.1859	0.0520	− 0.0231	− 0.1360	0.0897
40–44	0.2077*	0.0357	0.3796	− 0.1123	− 0.2389	0.0142	− 0.0433	− 0.1763	0.0896	− 0.0082	− 0.1487	0.1323	− 0.0686	− 0.1830	0.0459	− 0.0802	− 0.1983	0.0379
45–49	0.0851	− 0.1054	0.2755	− 0.1440	− 0.2951	0.0071	− 0.0915	− 0.2460	0.0629	0.0901	− 0.0334	0.2136	− 0.0330	− 0.1508	0.0849	− 0.0537	− 0.1779	0.0707
*Highest level of education*																		
No education	Ref																	
Primary	− 0.0576	− 0.1785	0.0633	− 0.1878 ***	− 0.2912	− 0.0843	− 0.1779 **	− 0.2790	− 0.0767	0.0290	− 0.0701	0.1281	0.0092	− 0.0810	0.0994	0.0451	− 0.0409	0.1311
Secondary	0.1087	− 0.0334	0.2509	-0.1782 **	− 0.2959	− 0.0606	− 0.1432 *	− 0.2641	− 0.0222	0.0398	− 0.0731	0.1526	− 0.0144	− 0.1119	0.0829	0.0426	− 0.0592	0.1443
Higher	0.1238	− 0.2193	0.4669	− 0.0225	− 0.2214	0.1764	− 0.4430**	− 0.7107	− 0.1753	− 0.0023	− 0.1969	0.1924	− 0.1934*	− 0.3541	− 0.0326	0.0789	− 0.1188	0.2766
*Sex of household head*																		
Male	Ref																	
Female	0.0188	− 0.1374	0.1750	0.0359	− 0.0680	0.1397	− 0.0216	− 0.1326	0.0893	0.0136	− 0.0851	0.1122	− 0.0035	− 0.0807	0.0730	0.0156	− 0.0720	0.1033
*Currently working*																		
No	Ref																	
Yes	0.0070	− 0.0713	0.0852	0.0441	− 0.0199	0.1080	0.0139	− 0.0666	0.0944	− 0.0833 *	− 0.1487	− 0.0179	− 0.0162	− 0.0623	0.0298	0.0204	− 0.0320	0.0729
*Currently in a union*																		
No	Ref																	
Yes	0.0138	− 0.1831	0.2106	0.1856 **	0.0641	0.3071	0.0994	− 0.0409	0.2400	− 0.1098	− 0.2701	0.0505	− 0.1235*	− 0.2457	− 0.0012	− 0.2022 ***	− 0.3094	− 0.0950
*Exposed to FP messages through media*																		
No	Ref																	
Yes	0.0598	− 0.0180	0.1376	− 0.0193	− 0.0850	0.0464	− 0.0538	− 0.1246	0.0170	− 0.0246	− 0.0825	0.0333	0.0409	− 0.0074	0.0893	− 0.0363	− 0.0955	0.0228
*Accessed contraceptive counselling*																		
No	Ref																	
Yes	− 0.1030 *	− 0.2052	− 0.0009	− 0.0400	− 0.1037	0.0236	− 0.1028 **	− 0.1725	− 0.0331	0.0470	− 0.0175	0.1116	0.0081	− 0.0420	0.0581	0.0527	− 0.0094	0.1147
*Health insurance coverage*																		
No	Ref																	
Yes	0.0814	− 0.1432	0.3059	0.1186	− 0.1527	0.3899	0.2093	− 0.1429	0.5615	− 0.1593 **	− 0.2527	− 0.0659	− 0.1574	− 0.3194	0.0047	− 0.1679	− 0.4182	0.0824
*Place of residence*																		
Urban	Ref																	
Rural	− 0.0919	− 0.2091	0.0253	− 0.0280	− 0.0945	0.0386	0.0301	− 0.0448	0.1049	0.0298	− 0.0339	0.0935	0.0499*	0.0007	0.0990	− 0.0241	− 0.0889	0.0408

*P < 0.05, **P < 0.01, ***P < *0.001*

Inequality in unmet need for contraception shows negative values of the EI indicating that it is the poor people that experience unmet need more compared to the rich. The inequality in unmet need for contraception largely remained the same (concentration index of approximately − 0.04) with an increase in 2013/14 (from a concentration index of − 0.0484 in 2007 to − 0.0940 in 2013/14). Inequality in unmet need was significantly influenced by religion, health insurance coverage and employment status in 2007; education, being in a union, place of residence in 2013/14; and being in a union in 2018. On the other hand, working (someone earning an income) and those with a health insurance coverage significantly increased inequality of unmet need for contraception towards the poor compared to those that were not working in 2007. Having higher education increased inequality by advantaging the wealthy: reducing unmet need among the wealthy in 2013/14. Women staying with their partners were associated with increased inequality in unmet need for contraception in 2013/14 and 2018; increasing inequality towards the poor compared to their counterparts that were not staying with partners. Staying in a rural area reduced inequality in unmet need for contraception in 2013/14; reducing the inequality among the poor compared to living in an urban area.

Supplementary material II and supplementary material III show the RIF-I-OLS decomposition of the different inequality indices (CI, ARCI, SRCI, WI) that have been estimated for the current use of modern contraception and unmet need for contraception. The different indices show similar direction of inequality as EI.

## Discussion

Contraceptive use among women of reproductive age in Zambia is increasing but sigificant inequalities in current use of modern contraception and unmet need for contraception are still evident. Current use of modern contraception is more concentrated among women in the wealthier half of the population than those in bottom half while the women in the bottom half bear most of the unmet need for contraception compared to their counterparts in the wealthier half of the population. However, the inequality in current use of modern contraception shows a reducing trend. Furthermore, our results show that inequality in modern contraceptive use is significantly driven by having no education, being in a union and low access to contraceptive counselling.

Current use of modern contraception is more concentrated among women in the wealthier half of the population. Similar findings have been reported from other sub-Saharan countries [17, 18]. Fentie et al [17] found that modern contraceptive use was pro-rich where the pooled EI for modern contraceptive use of 47 SSA countries was estimated to be 0.079. Our results show that the level of inequality in current use of modern contraception in Zambia (EI = 0.2046–0.1124) is clearly higher than that of the pooled SSA average estimate (EI = 0.079). This could explain why the fertility rate in Zambia remains high [56]. This calls for urgent and further intervention of government to tackle the causes of the inequality.

Our study shows that there is a gradually decreasing level of inequality in current use of modern contraception in Zambia during the period 2007–2018. The decrease could be a result of interventions targeting reproductive health that have been implemented in Zambia including results-based financing for reproductive maternal child adolescent health and nutrition [57], incorporation of contraception as part of maternal services in some health facilities and availability of a network of community health workers such is community health assistants, safe motherhood action groups, neighbourhood health committees. These interventions have been shown to increase access to contraception and encourage its utilisation [58]. Existence of such interventions could explain why place of residence (rural/urban) is not a significant determinant of inequality in current use of modern contraception. Based on our study, the decreasing inequality is partly driven by access to contraceptive counselling thus calling for more investment in increasing access to maternal health services where contraceptive counselling is an integrated part of services.

Although the inequality in current use of modern contraception has shown a steady gradual decline over time, the inequality in unmet need for contraception has not followed a similar trend, showing a very small and unsteady decline. This could signify that there is an increase in current use of modern contraception among the less well-off people generally but not as much to cover the demand for contraception within the group. It may also indicate that poorer individuals are increasingly desiring to limit or space child birth but experience barriers to access and use of contraception. This finding requires further investigation to identify and further understand inequality in population subgroups among the less well-off people that experience unmet need for contraception.

Furthermore, despite the decreasing overall level of inequality in unmet need for contraception, there was an increase in unmet need in 2013/14 (from EI = -0.0484 in 2007 to EI = -0.094 in 2013/14). This could have been a result of the collapse of the sector wide approach (SWAp) in Zambia in 2009 following corruption allegations. SWAp, initiated in Zambia in 1993, was a model where development partners pooled their funds into a central basket [59, 60] with the intention of strengthening health systems. With the implementation of SWAp, the MoH in collaboration with the development partners planned and allocated the funds based on population health needs, including provision of contraception to those that needed it. The collapse of the scheme may have reduced access to contraception and a reduction in quality of services for those in need thus increasing inequality.

Our results show that education level influences inequality in both current use of modern contraception and unmet need in Zambia. Women without an education were found to be disadvantaged regarding current use of modern contraception and unmet need. It is women with higher education that use modern contraception more and experience less unmet need for contraception compared to those that are not educated. In most cases it is the women that attain some education or higher that have more wealth thus the inequality in contraceptive use when compared with those without an education. This is similar to Makumbi et al. study in Uganda and other studies that reported that having higher education favoured wealthier half of the population [16, 61, 62]. Formal education is known to positively impact uptake of modern contraception [63]. However, our findings reveal that the influence of education on inequality was not significant in 2007. This could be explained by the absence of the comprehensive sexuality education (CSE) in schools at a time. Such educational interventions have been found to generally have a positive impact on contraceptive use [64] CSE was officially introduced in schools in Zambia by Ministry of Education around 2014 where students in grades 5–12 (10–19-year-olds) attended the sessions [65]. However, some schools had already started teaching sexual reproductive health (SRH) education before the official launch of the CSE framework as early as 2009 [66].

Our study finds that being in a union increases inequality in current use of modern contraception and unmet need for contraception making it more pro-rich. Being in a union reduces likelihood of modern contraceptive use as seen in many African countries [67]. This could be explained by the power dynamics between a couple. Men have been reported to wield higher decision-making power on whether to use contraception. The absence of financial autonomy and spousal discussion about contraception increases non-use of contraception [68]. Further, people that are not in union exhibit more autonomy and are likely to be more risk averse given the consequences of pregnancy without being in a union.

In Zambia, access to contraceptive counselling appears to significantly reduce inequalities in modern contraceptive use. This finding is supported by studies in India [69] and SSA [70] where contraceptive counselling was found to be positively associated with modern contraceptive use. Contraceptive counselling offers an interactive discussion between the health worker and client (woman of reproductive age) where the client receives information on contraception and can have questions on contraception answered. This process enables the client to select appropriate contraceptive methods that suit their needs and manage side effects [71]. The better the quality of the contraceptive counselling the more the chances a client will start using contraception [71, 72]. Therefore, based on our study, further investment in contraceptive counselling is warranted and its integration in maternal health clinics will kindle uptake of modern contraception and its continued use [9].

Exposure to family planning through media (radio, television and newspapers) has been reported to significantly reduce inequality in current use of modern contraception in many low- and middle-income countries [16–18, 61, 62]. Media has further been known to increase uptake of modern contraception [73, 74]. Surprisingly, our study does not support that finding. However, further investigation is warranted to understand the effectiveness and impact of the different media types on contraception uptake to inform policy on communication about contraception. This because considerable investments have been made in advocating for contraceptive use through media, radio, newspapers, and television by, among others, the Ministry of Health, non-governmental organisations, and civil society.

## Limitations

The definition of sexually active was limited to those that had had sex within the last 30 days before the interview. This may have left out persons that had sex outside this period. Furthermore, there is likely to be recall bias for questions that required the respondent to recall what had transpired within a year’s time. The use of contraception was self-reported, there could have been a risk of social desirability which may influence the findings.

We included women of reproductive age with the assumption that all these women have the potential of getting pregnant. However, it should be noted that not all of the women are fecund. Some of the women of reproductive age may be infecund and therefore may not need contraception for birth control. This may have implications on the results but given the low probability of being infecund, the implications on the results may be negligible.

## Conclusion

Our study shows that inequalities in current use of modern contraception have gradually decreased in Zambia and a limited decline in inequalities in unmet need for contraception. While this is encouraging, severe inequalities persist and are influenced by factors such as wealth status, education level and access to quality maternal health care services offering contraceptive counselling. To further reduce inequalities, the government should target people in union, and people without formal education. Continued investment to increase access to contraceptive counselling appears as an important intervention as it has exhibited a significant influence on contraceptive use in the Zambian setting.

## Supplementary Information

Additional file1 (DOCX 83 KB)

## Data Availability

No datasets were generated or analysed during the current study.

## Abbreviations

aOR: Adjusted Odds Ratio
ARCI: Attainment Relative Concentration Index
CCI: Corrected Concentration Index
CI: Concentration Index
COR: Crude Odds Ratio
CSE: Comprehensive Sexuality Education
DHS: Demographic Health Surveys
EI: Erreygers concentration Index
FP: Family Planning
NHIMA: National Health Insurance Management Authority
NHIS: National Health Insurance Scheme
RIF: Recentered Influence Function
SCT: Social Cognitive Theory
SDGs: Sustainable Development Goals
SRCI: Shortfall relative concentration index
SSA: Sub–Saharan Africa
STROBE: Strengthening the Reporting of Observational studies in Epidemiology
SWAP: Sector Wide Approach
WDW: Wagstaff, Doorslaer, and Watanabe
WI: Wagstaff Index
ZDHS: Zambia Demographic and Health Surveys
