# Supplementary material for: Inequality in modern contraceptive use and unmet need for contraception among women of reproductive age in Zambia. A trend and decomposition analysis 2007–2018
Source: Reprod Health. 2024 Dec 9;21:181. doi: 10.1186/s12978-024-01909-8 (PMC11629496; doi:10.1186/s12978-024-01909-8)
Supplement: Supplementary file 1 — Additional file1 (DOCX 83 KB) [file 12978_2024_1909_MOESM1_ESM.docx]

**SUPPLEMENTARY MATERIAL**

**Supplementary material I**

Supplement 1: Distribution of wealth status among the study participants (2007, 2013/14 and 2018)

**Supplementary material II**

Supplementary Material II: RIF-I-OLS Decomposition of the different concentration indices measuring inequality of modern contraceptive use

| Variable | 2007 | | | | 2013/14 | | | | 2018 | | | |
| --- | --- | --- | --- | --- | --- | --- | --- | --- | --- | --- | --- | --- |
|  | **CI** | **ARCI** | **SRCI** | **WI** | **CI** | **ARCI** | **SRCI** | **WI** | **CI** | **ARCI** | **SRCI** | **WI** |
| Age group |  |  |  |  |  |  |  |  |  |  |  |  |
| 15-19 | Ref |  |  |  |  |  |  |  |  |  |  |  |
| 20-24 | -0.0311 | -0.0311 | 0.0242 | -0.0069 | -0.0756* | -0.0756* | -0.0241 | -0.0997 | 0.0221 | 0.0221 | 0.0399 | 0.0620 |
| 25-29 | -0.0393 | -0.0393 | 0.0322 | -0.0070 | -0.0827* | -0.0827* | -0.0182 | -0.1010 | 0.0300 | 0.0300 | 0.0632* | 0.0932 |
| 30-34 | -0.0174 | -0.0174 | 0.0263 | 0.0089 | -0.0887** | -0.0887** | -0.0221 | -0.1109 | -0.0077 | -0.0077 | 0.0238 | 0.0162 |
| 35-39 | 0.0052 | 0.0052 | 0.0365 | 0.0416 | -0.0701 | -0.0701 | -0.0051 | -0.0752 | 0.0107 | 0.0107 | 0.0385 | 0.0492 |
| 40-44 | 0.1124 | 0.1124 | 0.0923* | 0.2046* | -0.0892* | -0.0892* | -0.0305 | -0.1197 | -0.0274 | -0.0274 | -0.0160 | -0.0434 |
| 45-49 | 0.0723 | 0.0723 | 0.0285 | 0.1008 | -0.0755 | -0.0755 | -0.0688 | -0.1442 | -0.0395 | -0.0395 | -0.0519 | -0.0914 |
| Highest level of education |  |  |  |  |  |  |  |  |  |  |  |  |
| No education | Ref |  |  |  |  |  |  |  |  |  |  |  |
| primary | -0.0469 | -0.0469 | -0.0201 | -0.0669 | -0.1167*** | -0.1167*** | -0.0757** | -0.1924*** | -0.1007*** | -0.1007*** | -0.0773** | -0.1781** |
| secondary | 0.0273 | 0.0273 | 0.0594* | 0.0867 | -0.1214*** | -0.1214*** | -0.0638* | -0.1851** | -0.0881** | -0.0881** | -0.0554 | -0.1435* |
| higher | 0.0336 | 0.0336 | 0.0668 | 0.1004 | -0.0460 | -0.0460 | 0.0155 | -0.0306 | -0.2247** | -0.2247** | -0.2184** | -0.4431** |
| Religion |  |  |  |  |  |  |  |  |  |  |  |  |
| Catholic | Ref |  |  |  |  |  |  |  |  |  |  |  |
| protestant | -0.0148 | -0.0148 | -0.0078 | -0.0226 | 0.0070 | 0.0070 | -0.0038 | 0.0032 | 0.0331 | 0.0331 | 0.0345 | 0.0677 |
| Muslim | -0.3222 | -0.3222 | -0.2226 | -0.5447 | 0.0016 | 0.0016 | -0.0047 | -0.0037 | -0.0529 | -0.0529 | -0.0030 | -0.0559 |
| other | 0.0888 | 0.0888 | 0.0396 | 0.1284 | 0.1313 | 0.1314 | 0.0559 | 0.1873 | 0.0574 | 0.0574 | 0.0505 | 0.1079 |
| Sex of household head |  |  |  |  |  |  |  |  |  |  |  |  |
| Male | Ref |  |  |  |  |  |  |  |  |  |  |  |
| female | 0.0052 | 0.0052 | 0.0101 | 0.0153 | 0.0214 | 0.0215 | 0.0151 | 0.0366 | -0.0050 | -0.0049 | -0.0165 | -0.0215 |
| Currently working |  |  |  |  |  |  |  |  |  |  |  |  |
| No | Ref |  |  |  |  |  |  |  |  |  |  |  |
| yes | 0.0130 | 0.0130 | -0.0002 | 0.0128 | 0.0290 | 0.0290 | 0.0166 | 0.0455 | 0.0049 | 0.0049 | 0.0090 | 0.0139 |
| Currently in a union |  |  |  |  |  |  |  |  |  |  |  |  |
| No | Ref |  |  |  |  |  |  |  |  |  |  |  |
| Yes | 0.0399 | 0.0399 | -0.0053 | 0.0345 | 0.0868** | 0.0868** | 0.0967** | 0.1835** | 0.04509 | 0.0451 | 0.0542 | 0.0993 |
| Exposed to FP messages through media |  |  |  |  |  |  |  |  |  |  |  |  |
| No | Ref |  |  |  |  |  |  |  |  |  |  |  |
| Yes | 0.0122 | 0.0122 | 0.0337* | 0.0459 | -0.0206 | -0.0206 | -0.0012 | -0.0218 | -0.0282 | -0.0282 | -0.0257 | -0.0538 |
| Accessed contraceptive counselling |  |  |  |  |  |  |  |  |  |  |  |  |
| No | Ref |  |  |  |  |  |  |  |  |  |  |  |
| Yes | -0.1300*** | -0.1301*** | -0.0196 | -0.1496** | -0.0441* | -0.0441* | -0.0014 | -0.0455 | -0.0621*** | -0.0621*** | -0.0408* | -0.1030** |
| Health insurance coverage |  |  |  |  |  |  |  |  |  |  |  |  |
| No | Ref |  |  |  |  |  |  |  |  |  |  |  |
| yes | 0.0135 | 0.0135 | 0.0469 | 0.0604 | 0.0613 | 0.0613 | 0.0573 | 0.1186 | 0.0986 | 0.0986 | 0.1106 | 0.2092 |
| Place of residence |  |  |  |  |  |  |  |  |  |  |  |  |
| Urban | Ref |  |  |  |  |  |  |  |  |  |  |  |
| rural | -0.0267 | -0.0267 | -0.0490 | -0.0757 | 0.0009 | 0.0009 | -0.0253 | -0.0243 | 0.0227 | 0.0227 | 0.0075 | 0.0302 |
| Index | 0.1406** | 0.1406** | 0.0803*** | 0.2209** | 0.0954** | 0.0954** | 0 .0865** | 0.1819** | 0 .0559*** | 0.0559*** | 0.0565*** | 0.1124*** |

**P < 0.05, **P < 0.01, ***P < 0.001*

*CI=concentration index, ARCI=attainment relative concentration index, SRCI=Shortfall relative concentration index, WI=Wagstaff Index*

**Supplementary material III**

Supplementary material III: RIF-I-OLS Decomposition of the different concentration indices measuring inequality of unmet need for contraceptive use

| Variables | 2007 | | | | 2013/14 | | | | 2018 | | | |
| --- | --- | --- | --- | --- | --- | --- | --- | --- | --- | --- | --- | --- |
|  | **CI** | **ARCI** | **SRCI** | **WI** | **CI** | **ARCI** | **SRCI** | **WI** | **CI** | **ARCI** | **SRCI** | **WI** |
| Age group |  |  |  |  |  |  |  |  |  |  |  |  |
| 15-19 | Ref |  |  |  |  |  |  |  |  |  |  |  |
| 20-24 | 0.0691 | 0.0692 | 0.0104 | 0.0796 | -0.0175 | -0.0175 | 0.0119 | -0.0057 | -0.0475 | -0.0475 | -0.0055 | -0.0530 |
| 25-29 | 0.0391 | 0.0391 | 0.0083 | 0.0474 | -0.0776 | -0.0776 | -0.0006 | -0.0782 | -0.1518* | -0.1519* | -0.0287 | -0.1805* |
| 30-34 | 0.0003 | 0.0003 | 0.0009 | 0.0012 | -0.0596 | -0.0596 | 0.0019 | -0.0577 | -0.1216 | -0.1216 | -0.0236 | -0.1452 |
| 35-39 | -0.0620 | -0.0620 | -0.0002 | -0.0622 | -0.1223 | -0.1223 | -0.0181 | -0.1404 | -0.0348 | -0.0348 | -0.0069 | -0.0417 |
| 40-44 | -0.0951 | -0.0951 | 0.0002 | -0.0949 | -0.1209 | -0.1209 | -0.0188 | -0.1397 | -0.1055 | -0.1056 | -0.0247 | -0.1303 |
| 45-49 | 0.0228 | 0.0228 | 0.0306 | 0.0534 | -0.1097 | -0.1097 | -0.0052 | -0.1149 | -0.0963 | -0.0964 | -0.0150 | -0.1114 |
| Highest level of education |  |  |  |  |  |  |  |  |  |  |  |  |
| No education | Ref |  |  |  |  |  |  |  |  |  |  |  |
| primary | 0.0430 | 0.0430 | 0.0087 | 0.0517 | -0.0029 | -0.0029 | 0.0039 | 0.0010 | 0.0468 | 0.0469 | 0.0147 | 0.0615 |
| secondary | 0.0604 | 0.0604 | 0.0119 | 0.0723 | -0.0374 | -0.0374 | -0.0031 | -0.0405 | 0.0363 | 0.0363 | 0.0143 | 0.0506 |
| higher | -0.0153 | -0.0153 | -0.0003 | -0.0156 | -0.2750** | -0.2750** | -0.0579* | -0.3329* | 0.0885 | 0.0885 | 0.0253 | 0.1137 |
| Religion |  |  |  |  |  |  |  |  |  |  |  |  |
| Catholic | Ref |  |  |  |  |  |  |  |  |  |  |  |
| protestant | 0.0560 | 0.0560 | 0.0111 | 0.0670 | 0.0496 | 0.0496 | 0.0105 | 0.0601 | -0.0238 | -0.0238 | -0.0076 | -0.0315 |
| Muslim | 0.9843 | 0.9843 | 0.1703* | 1.1546 | -0.0811 | -0.0811 | -0.0173 | -0.0984 | -0.0610 | -0.0610 | -0.0061 | -0.0671 |
| other | 0.3334 | 0.3334 | 0.0599 | 0.3933 | 0.2962 | 0.2964 | 0.0663 | 0.3627 | -0.0469 | -0.0469 | -0.0159 | -0.0628 |
| Sex of household head |  |  |  |  |  |  |  |  |  |  |  |  |
| Male | Ref |  |  |  |  |  |  |  |  |  |  |  |
| female | 0.0005 | 0.0005 | 0.0047 | 0.0052 | -0.0139 | -0.0139 | -0.0004 | -0.0143 | 0.0230 | 0.0230 | 0.0047 | 0.0276 |
| Currently working |  |  |  |  |  |  |  |  |  |  |  |  |
| No | Ref |  |  |  |  |  |  |  |  |  |  |  |
| yes | -0.1429** | -0.1429** | -0.0244* | -0.1673* | -0.0242 | -0.0242 | -0.0049 | -0.0290 | 0.0164 | 0.0164 | 0.0070 | 0.0233 |
| Currently in a union |  |  |  |  |  |  |  |  |  |  |  |  |
| No | Ref |  |  |  |  |  |  |  |  |  |  |  |
| Yes | -0.2533 | -0.2533 | -0.0301 | -0.2833 | -0.2626** | -0.2626** | -0.0306 | -0.2932** | -0.3297*** | -0.3297*** | -0.0585** | -0.3882*** |
| Exposed to FP messages through media |  |  |  |  |  |  |  |  |  |  |  |  |
| No | Ref |  |  |  |  |  |  |  |  |  |  |  |
| Yes | -0.0671 | -0.0671 | -0.0064 | -0.0735 | 0.0344 | 0.0344 | 0.0140 | 0.0484 | -0.0564 | -0.0564 | -0.0107 | -0.0670 |
| Accessed contraceptive counselling |  |  |  |  |  |  |  |  |  |  |  |  |
| No | Ref |  |  |  |  |  |  |  |  |  |  |  |
| Yes | 0.0645 | 0.0645 | 0.0143 | 0.0788 | -0.0019 | -0.0019 | 0.0034 | 0.0015 | 0.0630 | 0.0630 | 0.0166 | 0.0796 |
| Health insurance coverage |  |  |  |  |  |  |  |  |  |  |  |  |
| No | Ref |  |  |  |  |  |  |  |  |  |  |  |
| Yes | -0.3008** | -0.3008** | -0.0457** | -0.3465** | -0.1940 | -0.1940 | -0.0493 | -0.2434 | -0.2277 | -0.2277 | -0.0514 | -0.2791 |
| Place of residence |  |  |  |  |  |  |  |  |  |  |  |  |
| Urban | Ref |  |  |  |  |  |  |  |  |  |  |  |
| Rural | 0.0632 | 0.0632 | 0.0083 | 0.0715 | 0.0866** | 0.0865** | 0.0138 | 0.1003* | -0.0241 | -0.0241 | -0.0079 | -0.0320 |
| Index | -0.0770*** | -0.0770*** | -0.0143*** | -0.0914*** | -0.1113*** | -0.1113*** | -0.0298*** | -0.1411*** | -0.0524*** | -0.0524*** | -0.0134** | -0.0658*** |

**P < 0.05, **P < 0.01, ***P < 0.001*

*CI=concentration index, ARCI=attainment relative concentration index, SRCI=Shortfall relative concentration index, WI=Wagstaff Index*
